# A Review on Treatment Approaches for Chronic Low Back Pain via Mulligans Movement With Mobilization and Physical Therapy

**DOI:** 10.7759/cureus.28127

**Published:** 2022-08-18

**Authors:** Neha Chitale, Deepali S Patil, Pratik Phansopkar, Aditi Joshi

**Affiliations:** 1 Musculoskeletal Physiotherapy, Datta Meghe Institute of Medical Sciences, Wardha, IND; 2 Physiotherapy, Ravi Nair College of Physiotherapy, Wardha, IND; 3 Musculoskeletal Physiotherapy, Ravi Nair Physiotherapy College, Datta Meghe Institute of Medical Sciences, Wardha, IND

**Keywords:** physical therapy, chronic low back pain, rehabilitation, mulligan mobilization, low back pain

## Abstract

Low back pain is a leading cause of functional disability. Low back pain is a problem that every one of all age groups faces and there are various methods used to correct low back pain. Manual therapy is a specialized area in physiotherapy which manages neuromuscular pain. Manual therapy techniques include the Maitland mobilization technique, Kaltenborn mobilization technique, Mulligan technique, Active release technique, and many more. Manual therapy mainly works on arthrokinematics and osteokinematics of the joint. It is one of the main strategies used to manage low back pain. Low back pain can be because by spinal dysfunction, Sacroiliac dysfunction, muscle pathology, or any ligamentous strain. The cause of the low back pain should be identified and treated accordingly. Articles using keywords - chronic low back pain, Mulligan, and non-specific low back pain were searched on the database of Pubmed and Scopus a total of 30 articles from the timeframe of 2011 to 2021 were included in the study. From this review, we can conclude that Mulligan mobilization with movement is an effective way to reduce low back pain. Further studies need to be conducted to check the effectiveness of manual therapy techniques in chronic low back pain.

## Introduction and background

Pain in the back region that lasts for more than three months is called chronic lower back pain (CLBP). Lower‐back pain (LBP) is seen in all age groups. Half of the people encounter back pain in a year and nearly three-fourths of the population account for LBP in their lifetimes [1]. A 2015 study showed the global prevalence of LBP as 7.3%. Activities that are required for daily living are affected because of LBP. The significance of disability is created because of LBP. The lumbosacral, sacroiliac and lumbar regions are the main regions of LBP [2]. LBP can be because of some specific cause like a fracture it is persistent or can be because of some unknown nociceptive source called non-specific LBP [3-5]. LBP has a high prevalence and needs special attention while treatment. Evidence exists stating the effectiveness of exercise therapy on LBP, while very few of literature gives input on the effect of Manual therapy on LBP.

Manual therapy is a specialized area in physiotherapy which manages neuromuscular pain. Manual therapy techniques include the Maitland mobilization technique, Kaltenborn mobilization technique, Mulligan technique [6], active release technique, and many more. Manual therapy mainly works on arthrokinematics and osteokinematics of the joint. It is one of the main strategies used to manage LBP.

Mulligan is a technique in manual therapy which says that arthrokinematics and osteokinematics of the joint can be bought to normal by performing mobilization with movement, as sometimes the issue cannot be corrected just by mobilization in a stationary position. Mulligan proposed that there are some positional faults in the joint created because of injury or prolonged use and these positional faults leads to pain and reduced range of motion. Mulligan's concept helps in correcting the postural faults by performing mobilization with movement [7].

LBP is a problem that everyone of all age groups faces and there are various methods used to correct LBP. Manual therapy methods include- mulligan mobilization, Kaltenborn mobilization, Mckenzie technique, active release technique, and many more. LBP can be because by spinal dysfunction, sacroiliac dysfunction, muscle pathology, or any ligamentous strain [8]. The cause of the LBP should be identified and treated accordingly.

Pathophysiology of LBP

The muscles of the trunk and the spinal operate in collaboration to move the spine. Primary movements include flexion, extension, lateralization, and rotation [9]. Flexion/extension refers to rotations in the sagittal plane, whereas lateral bending refers to rotations in the frontal plane. Axial rotation is a rotation of the spine around its vertical axis. Facet joints are critical in maintaining lumbar stability during forwarding flexion. The facets bear considerable loads during axial rotation, allowing the resulting applied force to fall adjacent to the posterior edge of the articulating surface. Any pathology to the facet joint or intervertebral disc leads to LBP. The spine's stabilization is restricted in LBP to sustain intervertebral neutral zones, leading to pain, extreme deformity, and neurological dysfunction, resulting in the spine becoming more unstable and excessive movements, which lead to muscle stretch, nervous compression, and inflammation, that all contribute to LBP.

Spinal pain can be caused by a multitude of factors, including mechanical, structural, functional, psychological, and neuromuscular dysfunctions. MTrPs are most common in quadratus lumborum and iliocostalis lumborum muscles, and a higher number of active MTrPs was found linked with increased pain intensity [10]. They are characterized by muscle stiffness, tenderness, and pain that radiates to other regions, a condition known as referred pain [11]. The general risks are events of trauma factors ergonomic, factors of structure, and factors of the system [12]. They may also occur as a result of athletic training and muscle strain, or they can be due to an underlying physical condition. They are typically identified by inspecting a muscle for knots or small regions of muscle spasm within a tense band of muscle that is tender and inflicts referred pain. Trigger points are divided into two categories: active and latent.

Prevention of LBP

LBP can be experienced in any age group. Risk factors include improper long sitting posture, prolonged standing, and improper lifting techniques. Considering this, LBP can be prevented by improving posture using mirror feedback, improving the core muscles' strength, and using proper lifting techniques as well as during flexibility training [4].

## Review

Non-specific LBP (NSLBP)

The type of LBP where the cause is not known is referred to as NSLBP. There is no specific disease of anatomical or biomechanical alteration in the back region but the pain persists. Mechanical aspects were long considered to play a part in LBP. Using the Bradford-Hill causation criteria, there is no independent association between posture which is uncomfortable, prolonged standing or walking, handling, pulling or pushing twisting, or the way things are carried. Mechanical LBP is described as back pain resulting from increased tension and strain in the spinal column and muscles, usually due to unhealthy behaviors, such as poor posture, inappropriate sitting, and improper bending and lifting activity. Mechanically LBP in the back is characterized by discomfort that aggravates movement and improves rest [13]. Non-specific LBP mainly occurs because of decreased strength in the back and core muscles or due to poor postural control and sacroiliac displacement. Muscle activation is reduced in such cases which leads to pain and restricted range of motion (ROM), as the ROM is restricted activities of daily living also become difficult to perform leading to disability.

Vlaeyen et al. reviewed LBP and stated that LBP is frequently characterized by muscle tension stiffness and pain situated in the gluteal and LB region. Secondary to the SI joint displacement and tendinitis of the SI joint stabilizers, including the quadratus lumborum, the glutei, the piriformis, the iliopsoas, and others. A vast majority of people with back pain have nonspecific pain, with no identifiable root pathology or nociceptive contributor. Back pain has a serious adverse influence on the quality of life of those who suffer from it. Back pain raises the risk of disability, and the negative effect on QOL worsens as the pain persists. It is correlated with fear and worries, especially regarding one's (sense of) self and social interactions, and it is aggravated when pain lasts longer than anticipated [14]. A study was done on LBP by Violante et al. and described LBP as “pain, muscle tension, or stiffness localized in the gluteal region, leg region, and sacroiliac region." Clinical recommendations often identify two types of LBP: NSLBP, which is described as a condition caused by no recognized specific pathology, and specific LBP, which is caused by a recognized known particular pathology [15]. Furthermore, although pain and impairment can continue for a longer period in some situations, LBP is once again categorized as acute, subacute, and chronic in which acute is the pain which is lasting less than six weeks, and sub-acute is between six and 12 weeks and chronic is more than 12 weeks. Identifying the type of LBP, if it is acute/Subacute/chronic and specific or nonspecific is important for epidemiological research as well as a clinical treatment.

Sharan et al. in the year of 2014 studied myofascial LBP (MLBP) treatment and stated that the position of muscular factors, especially myofascial pain syndrome (MPS). MPS seems to be a very frequent cause of LBP, is highly treatable, and therefore should be recognized as a major influence throughout all cases of LBP. Exercises are effective in both the sub-acute and chronic phases of MLBP. Physical modalities, as well as strength and stretching techniques, are beneficial. Resistance exercises minimized pain while still enhancing bone health by increasing bone mineral density and correcting particular aging causes in muscles, thus maintaining the muscles healthy. During training, biofeedback serves as motivation and a feedback source, encouraging them to enhance their results to the target stage [16].

Mulligan mobilization with movement

Mulligan's concept says that pain is due to a minor positional fault to the joint which is leading to the restriction. Postural fault leads to biomechanical alteration leading to pain [17]. In Mulligan mobilization sustained natural apophyseal glide is given for the spine and extremities movement with mobilization is given. Passive accessory movement is given to the spinous process and transverse process and the patient is asked to perform movements like flexion, and extension. The principles of Mulligan mobilization are that the movement should be pain-free [18].

Hussien et al. did a study on the Effect of the Mulligan concept of lumbar sustained natural apophyseal glide (SNAG) on Chronic NSLBP. Forty-two participants were selected who had NSLBP and divided into two groups randomly. Conventional physiotherapy treatment, which included strengthening and stretching of the muscles, was given to both groups whereas the experimental group received Mulligan’s concept SNAG at the level of the spine where the maximum affection was present. The treatment was given for a month with three sessions per week. The result was noted using an isokinetic dynamometer, with pain and functional disability as the outcome measures. Before and after the treatment all three outcomes were recorded. Post statistical analysis improvement was seen in both groups. SNAG along with conventional programs as a treatment approach for chronic nonspecific LBP gave better results in terms of pain reduction and improvement in functions [7,19].

Namnaqani et al. conducted a systematic review on the effect of the Mckenzie method in comparison with manual therapy. They included five trials in the study. The primary outcome was pain along with disability. They concluded that Mckenzie is a better treatment approach to reduce the pain in short term [20]. McKenzie works on the principle of peripheralization and centralization and the Mckenzie treatment approach depends on individual pre-treatment assessment [21].

McCaskey et al. conducted a systematic review in which they included 18 studies of proprioceptive exercises on chronic neck and LBP. They considered Pain and functional independence as outcome measures. They concluded that no consistent benefit was seen in proprioceptive exercises on chronic neck and LBP [22]. Bialosky et al. checked the effect of spinal manipulation therapy on LBP. A total of 110 participants were included in the study, numeric pain rating scale (NPRS) for pain and Oswestry Disability Index for functional disability were taken as an outcome. The study concluded that spinal manipulation therapy is effective [23].

Rajfur et al. conducted a comparative pilot study to check the effect of electrotherapy on CLBP. In the study, they included conventional transcutaneous electrical nerve stimulation (TENS), acupuncture TENS, high-voltage electrical stimulation, interferential current, and diadynamic current as treatment approaches. They concluded that interferential current has a significant effect in reducing pain as it penetrates deeper while TENS and high-voltage correct were also helpful but not effective [24]. Peripheral and central stimulation are non-invasive techniques. Central stimulation is termed transcranial direct current stimulation. Both central and peripheral stimulation has analgesic effects. So, can be helpful in reducing CLBP [25].

Li et al. conducted a systematic review to check the effect of kinesio-taping on CLBP. They included a total of 10 articles and pain and functional disability were assessed. No superior effect of kinesio taping was seen over placebo taping [26].

Peerachotikphun et al. reviewed the effect of hydrotherapy on LBP. Water has the properties of viscosity, surface tension, hydrostatic pressure, and buoyancy help in stretching and strengthening muscles. Water reduces the risk of injury and hence can be used as an alternative for land exercises [27]. Different types of exercise protocols can be used for the treatment of CLBP. Pain neurophysiology education is educating the patient about pain and cognitive misalignment, which helps in relieving CLBP (Table 1) [28].

**Table 1:** Summary of studies reviewed on chronic low back pain

Author	Study Type	Study Sample	Intervention	Outcome	Intervention period	Result	Analysis
Namnaqani, et al. [20]	Systematic Review	160 subjects with chronic low back pain.	McKenzie and Manual Therapy	Visual Analogue Scale (VAS), Numeric Pain Rating Scale (NPRS), Symptom Bothersomeness scale, McGill Pain Questionnaire, Oswestry Disability index	4 times a week over 6 months	At 6 months follow-up, there were several improvements seen in both groups, but it was reported that a significant difference was seen in the McKenzie method group.	McKenzie is a successful treatment for the short term and hence enhances the performance providing long-term effect.
Peterson, et al. [21]	A randomized control trail.	350	McKenzie, Spine manipulations	Roland Morris Questionnaire, VAS, Oswestry Disability Index (ODI)	3 sessions per week for 8 weeks	Both the treatments showed improvement but after 2 months of follow-up, the McKenzie group was superior to manipulations.	McKenzie works on peripheralization and centralization. McKenzie is an effective way.
McCaskey, et al. [22].	A systematic Review	80	Proprioceptive exercises, stretching, strengthening, and endurance training	NPRS, ODI, Neck Disability Index (NDI)	3 sessions per week for 8 weeks.	The addition of proprioceptive exercises with traditional exercises leads to much improvement in pain and functional status of subjects.	There is no consistent benefit of proprioceptive exercises.
Biolosky, et al. [23]	Randomized Control Trail	110	Spinal Manipulation Therapy, Pain threshold, TENS modality.	Pressure Algometer, VAS, pain-centered outcome questionnaire, ODI	Twice a week, 15 minutes for 1 month.	A significant improvement in pain was observed after spinal manipulation therapy was given to all participants and improvement in functional activities also.	Spinal manipulation technique has an effect on pain sensitivity which is related to central sensitization.
Rajfur, et al. [24]	Comparitive Clinical Pilot study.	127	Conventional TENS, Accupunture-TENS, High-voltage electrical stimulation, Interferential current stimulation, Diadynamic current	VAS, ODI, Roland-Morris Disability Questionnaire		Usage of electrical stimulation with interferential current resulted in significant and more efficient elimination of pain and improvement of functional ability of patients suffering from low back pain.	Electrotherapy is a effective way to reduce pain and disability as it works on the pain gait mechanism.
Hazime, et al. [25].	Randomized control Trail	92	Transcranial direct current stimulation, transcutaneous electrical nerve stimulation	NPRS, ODI	12 sessions over some time four weeks.		The study was conducted to see the effect of peripheral and central stimulation as they both have analgesic effect.
Yuejie, et al. [26].	Meta Analysis	60	Kinesiotape, stretching, strengthening exercises.	VAS,ODI	Twice a week for 6 weeks	The effect of Kinesio tape alone was less than when added to the physical therapy exercise protocol.	Kinesio taping provides kinaesthetic feedback helping in proper posture maintenance.
Hussien, et al. [7].	Randomized Control Trail	42	Stretching exercises, strengthening exercises, SNAG mulligan concept	Isokinetic Dynamometer, VAS, Oswestry Disability Index.	Thrice a week over 1 month	Adding SNAG to conventional therapy resulted in higher improvement in terms of postural error, pain, and functional ability.	SNAG helps in reducing micro mal alignment and hence reduces the pain.
Sawant, et al. [27].	Randomized Control Trail	30	Extensor stretching exercises, and strengthening exercises for the back and core.	VAS, Modified Oswestry Disability Index .	30 minutes 5 times a week for 4 weeks	There was a significant improvement in subjects who underwent conventional therapy along with hydrotherapy.	Aquatic environment reduces the body weight inside the water due to buoyancy making the activities easy to perform and hence reducing pain.
Pires, et al. [28].	Randomized Control trail	62	Aquatic exercises, range of motion exercises, pain therapy	VAS, Quebec Back Pain Disability Scale, Tampa Scale of Kinesiophobia	12 sessions for 6 weeks	This study found that Adding pain neurophysiology education with aquatic exercises was helpful for all participants.	Pain can be significantly reduced using aquatic therapy. Because of the properties of water.

Seo et al. conducted a pilot RCT on the Effects of Mulligan Mobilization and Low-Level Laser Therapy (LLLT) on Physical Disability, Pain, and ROM in Patients with CLBP. In the study a total of 49 samples were included, they were randomly divided into three groups Group A received SNAG with LLLT, Group B received SNAG and Group C was the control group. Group A received SNAGs for 10 min, LLLT for 10 min, and electrotherapy for 10 min. Group B received SNAGs for 10 min and electrotherapy for 20 min while Group C received 30 min of electrotherapy. Participants received the treatment three times a week for four weeks. The pain was assessed using a visual analog scale, the range of motion of the lumbar spine was assessed using the modified-modified Schober's test and the Roland Morris disability questionnaire was used for physical disability. The study concluded that there is a significant effect of combined treatment of SNAGs and LLLT in terms of reduction in pain and disability to treat chronic pain [3].

Bontrupa et al. studied LBP and the effect of prolonged sitting and a sedentary lifestyle and noticed there was a stronger correlation between the behavior of sitting and CLBP than with disability, due to people with CLBP having an awareness level high of sitting positions which is pain-free and activities which provoking pain activities compared to individuals affected by acute pain. There was a slight correlation between overall behavior of sitting and individuals that reported CLBP and functional disability which was because of pain [29]. In 2018, Kothawale and Rao did a single intervention, comparative study on the effects of PRT versus ART on hamstrings tightness in asymptomatic females 18-30 years and illustrate the cycle of injury, It showed that tight muscles are more prone to injury due to tension in the structure of myofascial. ART serves to relieve as well as release the muscle tension, tendons, and the fascia that covers them by breaking up adhesions and retaining soft-tissue integrity. Is this accomplished through having contact with adhesion while shortened muscles and extending the muscle across its fibers further break up the adhesion? Muscles, tendons, and ligaments will function more freely, relieving pressure on the nerve and resolving pain. It was learned that ART is efficient in immediately reducing hamstring tightness [30].

A systemic review on the Effects of Exercise and Physical Activity (PA) on Non-Specific CLBP was conducted in 2016 wherein they learned that back pain would be classified as either specific or non-specific. NSLBP is identified when the root of the pain is unknown, whereas specific back pain is induced by a specific cause, such as an infection or a fracture. The main popular version of back pain is NSLBP. PAs enhance blood supply to the back, which is essential for the healing of soft tissues in the back. PA in the form of everyday tasks has been documented as significant in facilitating the recovery of acute and NSCLBP. CLBP limits trunk mobility to minimize lumbosacral pain, yet that reduces core strength and enhances lumbar instability, leading to LBP. Exercises to activate the deep abdominal muscles, along with the superficial muscles, transversus abdominis muscle, and multifidus, become crucial. Core stability methods are said to effectively minimize CLBP, as has a muscular strength program [31].

In 2022, a study was conducted on MFR, MET, and Stretching of Quadratus Lumborum Muscle in Subjects with NSLBP they included 35 participants and divided into two groups. The treatment was given for two weeks. They concluded that MFR stretching and MET when given in combination are effective in the treatment of subjects with NSLBP, As they help treat the tendinitis of the sacroiliac joint stabilizer muscles [32]. Çirak et al. researched to check the effect of SNAG on muscles in the lumbar region in terms of stiffness. Subjects with NSLBP were given Mulligan Lumbar SNAG. They aimed to see the effect of Mulligan SNAG on the stiffness of muscles in subjects with NSLBP using ultrasound waves and the effect on pain and disability was seen. They concluded that the subjects in the SNAG group had improved compared to the control group [33].

Mistry et al. performed a study on the effectiveness of proprioceptive neuromuscular facilitation and active release technique on the flexibility of hamstring muscle in Subjects with CLBP in which they found that the purpose of ART, like all soft tissue approaches, would be to eliminate these “adhesions” thus preventing the injury cycle as the tissue is transitioned from a muscle which is shortened to a completely lengthened position while the touch hand maintains tension longitudinally around the soft tissue ﬁbers. ART is being used to regain uninhibited soft tissue motion, loosen entrapped nerves, and return maximum soft tissue function. PNF Modified Hold Relax and ART both enhance hamstring flexibility thereby decreasing pain and disability over time [34]. A systematic review with Mackenzie and Manual therapy as an intervention was performed by Mamnaqani et al. they concluded that at six months of follow-up, there were several improvements seen in both groups, but it was reported that a significant difference was seen in the McKenzie method group. Mulligan Concept SNAG improves the range of motion of the spine, it also reduces the pain by reducing any disruption in the facet joint because of which there is a significant improvement in sympathetic activity. Mulligan’s SNAG when used in the treatment of LBP also affects reducing the muscle spasm and stiffness as it stimulates the autonomic nervous system. Pain threshold has also been reported to reduce, which leads to increased pain receptor exposure to neurogenic inflammation. Shreds of evidence have suggested changes in substantia alba and substantia grisea [33].

Material handling has traditionally been linked to LBP manually. Manual material handling instead of a single risk factor is a typical standard that can lead to spine overload (which is, ultimately, the risk factor for low-back pain). The treatment of big structures can result in spinal overload, especially when used in an unusual posture or a trunk bending and twisting: since managers maintain such a common duty, it is not unexpected that the word handling and manual lifting of the materials are often used in exchange. Moving and lifting heavy things might potentially create a spinal overload [15]. LBP is associated with substantial reductions in health and quality of life, as an incapacitating condition (HRQoL). Therefore, the evaluation of therapies or programs for LBP and the selection to allocate resources are necessary through reliable and valid HRQoL measures. Using disease-based or generic questionnaires, HRQoL may typically be assessed. Generic instruments can, in turn, be divided into preference rather than preference. Generic preferred measures mainly benefit from their broad range of health aspects, allowing comparisons of various diseases, treatments, and health programs.

## Conclusions

LBP is a common condition and all age groups are facing it. Pain can be due to various causes and the line of treatment depends upon the cause of pain. General strengthening exercises have proved to be effective in reducing LBP. There are many studies conducted to check the efficacy of various treatment modalities on LBP including the McKenzie technique, mulligan mobilization as well as aquatic therapy. In this review, we found out that LBP is a condition everyone is facing. Mulligan mobilization is a better treatment approach over other manual therapy techniques to reduce chronic LBP. Pain stiffness and disability were the points considered while concluding.

Apart from Mulligan mobilization back strengthening exercises as well as core strengthening exercises also help in reducing pain stiffness and increasing the range of motion further reducing the LBP. LBP affects the quality of life and to maintain or improve the quality of life strengthening the muscles plays an important role. This review gives a brief about the treatment methods available for treating LBP clinically.
